# The HydroColor App: Above Water Measurements of Remote Sensing Reflectance and Turbidity Using a Smartphone Camera

**DOI:** 10.3390/s18010256

**Published:** 2018-01-16

**Authors:** Thomas Leeuw, Emmanuel Boss

**Affiliations:** 1 Sequoia Scientific, Inc., 2700 Richards Road, Suite 107, Bellevue, WA 98005, USA; 2 School of Marine Sciences, University of Maine, 458 Aubert Hall, Orono, ME 04469, USA; emmanuel.boss@maine.edu

**Keywords:** reflectance, turbidity, mobile, smartphone, camera, crowdsourcing, water-quality

## Abstract

HydroColor is a mobile application that utilizes a smartphone’s camera and auxiliary sensors to measure the remote sensing reflectance of natural water bodies. HydroColor uses the smartphone’s digital camera as a three-band radiometer. Users are directed by the application to collect a series of three images. These images are used to calculate the remote sensing reflectance in the red, green, and blue broad wavelength bands. As with satellite measurements, the reflectance can be inverted to estimate the concentration of absorbing and scattering substances in the water, which are predominately composed of suspended sediment, chlorophyll, and dissolved organic matter. This publication describes the measurement method and investigates the precision of HydroColor’s reflectance and turbidity estimates compared to commercial instruments. It is shown that HydroColor can measure the remote sensing reflectance to within 26% of a precision radiometer and turbidity within 24% of a portable turbidimeter. HydroColor distinguishes itself from other water quality camera methods in that its operation is based on radiometric measurements instead of image color. HydroColor is one of the few mobile applications to use a smartphone as a completely objective sensor, as opposed to subjective user observations or color matching using the human eye. This makes HydroColor a powerful tool for crowdsourcing of aquatic optical data.

## 1. Introduction

The prevalence of sensor rich smartphones and high-speed wireless data connections has opened the door for low cost, large-scale data collection. Several studies have already demonstrated how smartphone sensors can be used to crowdsource atmospheric aerosol concentration, air temperature, seismic activity, and weather [1,2,3,4,5].

In the oceanographic and limnological communities, the use of citizen science as a method of data collection is not well established. This is partially due to the lack of tools that are both robust and easy for citizen scientists to acquire and use. Many low cost tools for studying water quality require a significant initial time commitment (e.g., building a waterproof housing, constructing a Secchi disk, soldering electrical components, etc.). Our aim was to produce a simple yet robust mobile application that can be used for crowdsourcing water quality data. Ideally, the application should require a low commitment, yet produce results comparable to traditional water quality methods. The result was the HydroColor water quality application (available for iOS and Android devices).

HydroColor uses a smartphone digital camera as a three-band radiometer. The light intensity measured in a series of three images is used to compute the above water remote sensing reflectance (*R_rs_*) in the red, green, and blue color channels of the camera. We show that a camera can be a viable light sensor, and that smartphones are particularly well suited for this application. The reflectance measured by HydroColor was directly compared with remote sensing reflectance measurements from precision radiometers. Additionally, the remote sensing reflectance was used to estimate water turbidity. The reflectance measured by HydroColor also has the potential to provide information on chlorophyll and dissolved organic matter.

Other studies have demonstrated working methods to measure water quality using a digital camera [6,7]. These previous studies used a digital camera fitted to a tube that extended below the surface of the water. The color of the images was used to estimate the concentration of chlorophyll and dissolved organic matter. The HydroColor application distinguishes itself from the previous studies in that it measures actual radiometric quantities instead of analyzing image color. Although this research focuses primarily on the HydroColor application, the methods described in this paper could be applied to virtually any digital camera.

The advantage of a reflectance based measurement is the direct comparability to measurements routinely made from other remote sensing platforms. Satellite remote sensing in particular has become an invaluable tool for oceanographers and limnologists [8]. HydroColor can complement these measurements by providing comparable ground based measurements. Therefore, the data produced by HydroColor can be aligned with the existing scientific goals of several earth observation programs.

## 2. Materials and Methods

The HydroColor application requires a smartphone, a photographer’s 18% reflectance gray card, and access to optically deep water. Optically deep refers to a water depth where light reflection from the bottom does not influence the water leaving radiance. The smartphone must be equipped with a digital color camera, compass, and gyroscope. An internal Global Positioning System (GPS) and a data connection are beneficial but not required. These components are standard in all modern smartphones.

The digital camera is the component used for sensing the environment. The other required sensors are used for auxiliary data that aids in the collection of images. The resolution of the camera is not critical to the radiance measurement. However, the resolution will have an effect on the field of view seen by the HydroColor application. The internal Bayer filter contained in the color camera consists of an alternating pattern of red, green, and blue (RGB) filters. This allows the camera to measure light intensity in broadband RGB. 

A compass and gyroscope are needed to orient the camera in the correct position to capture images. These sensors are present in most modern smartphones. The azimuth and zenith angles of the camera are critical for HydroColor to properly remove surface reflected light that enters the camera. The HydroColor application uses the compass and gyroscope to create an interactive display that guides the user to the correct azimuth and zenith angles for each image. The angular requirements of the images are described in the next section. 

An internal GPS and data connection allows the smartphone to automatically acquire the user’s latitude and longitude. The user’s location and the Coordinated Universal Time (UTC) are used to determine the position of the sun. The sun position is used to direct the user to the correct azimuth angle for image collection. The user also has the option to enter their GPS coordinates manually. Thus, an internal GPS or data connection is not required to use the application.

HydroColor requires a surface of known reflectance to measure the downwelling irradiance. We found that a photographer’s 18% reflectance gray card was a suitable low cost reflectance standard. Gray cards are widely available online and in photography shops, typically for less than $10 USD. Several previous studies have also used gray cards as reflectance standards [9,10]. While the standard gray card is 18% reflectance across the visible spectrum [11], cards with other reflectance values are available. Therefore, it is important to ensure the gray card reflectance is labeled as 18% before using it with the HydroColor application. In custom applications of this method, any surface of known reflectance can be used provided it is a Lambertian reflector and its reflectance value is used in the computation of remote sensing reflectance. 

### 2.1. Image Capture

To clarify the image capture procedure, the equation used to calculate above water remote sensing reflectance must be introduced. The following equation from Mobley [12] is commonly used to determine the remote sensing reflectance from above water radiometers:

(1) $$R_{rs}=\;\frac{L_{t}-\rho L_{s}}{\frac{\pi }{R_{ref}}L_{c}}$$

where *L_t_* is the radiance leaving the water surface, *L_s_* is the sky radiance, *R_ref_* is the irradiance reflectance of a reflectance standard (0.18, for the 18% gray card used here), *L_c_* is the measured radiance leaving the reflectance standard, and *ρ* is the sea surface reflectance factor. For clarity, the angular and spectral dependence of these quantities has been dropped (see Table 1 for list of abbreviations and notations).

Note that the denominator is equal to the downwelling irradiance (E_d_). By measuring the radiance of a reflectance standard, the downwelling irradiance can be measured without the use of a cosine collector, assuming the standard is a Lambertian reflector. One radiometer can be used to make all the required light measurements in Equation (1). The benefit of using one radiometer is that any scaling factor or multiplicative error cancels out in Equation (1). Therefore, the radiometer needs no absolute calibration. This is an important consequence of Equation (1), and is the key to HydroColor’s ability to measure remote sensing reflectance without the camera being calibrated to measure radiance.

The value for *ρ* has been determined using HydroLight, which is a radiative transfer software used for modeling above and below water radiance distributions. The specific value of *ρ* varies with environmental conditions and observation angle; however, the HydroColor application is programmed assuming a sun in a clear, cloudless sky. For custom applications, other sky conditions could be used, and the value of *ρ* adjusted accordingly. To minimize the amount of surface reflectance, and thus *ρ*, the water surface radiance (*L_t_*) should be measured at an azimuth angle 135° from the sun and 40° from nadir. Under ideal conditions, the value of *ρ* is approximately 0.028 for these angles. See Mobley [12,13] for an in-depth discussion of the dependence of *ρ* on viewing angle and sky conditions. 

**Figure 1:**
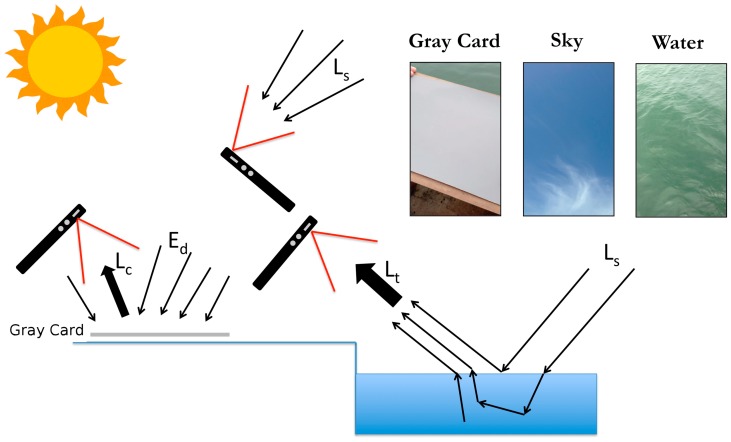
Example of three images collected with HydroColor and the optical features captured in each image. The gray card image is captured at 40° from nadir and 135° from the sun. The sky image is captured at 130° from nadir and 135° degrees from the plane of the sun. The water image is captured at 40° from nadir and 135° degrees from the plane of the sun.

To fulfill the radiance requirements of Equation (1), three images must be captured. An image of a gray card is needed to determine the card radiance (*L_c_*), an image of the sky is needed determine the sky radiance (*L_s_*), and lastly, an image of the water is needed determine the radiance from the water surface (*L_t_*) (Figure 1). Care should be taken to avoid capturing a water or gray card image that is shaded by a boat, dock or any other structure. Additional recommendations for collecting HydroColor measures can be found in Appendix A. 

Even though the surface reflection cannot be eliminated, it can be significantly reduced by collecting the water image at an angle of 135° from the sun and 40° from nadir. HydroColor collects a water image at these specific angles by taking advantage of the smartphone’s clock, GPS, compass, and gyroscope. 

HydroColor uses the current GPS coordinates and the current UTC time to determine the position of the sun in the sky. HydroColor puts this information into a simple sun position model that runs onboard the phone [14]. HydroColor uses the resulting sun azimuth angle to create a compass display. The compass display guides users to point the phone ±135° from the plane of the sun. The sun zenith angle is not used; however, it is saved along with the rest of the data. It can be used later for quality control. 

The gyroscope measures pitch, yaw, and roll of the phone. HydroColor uses the pitch and roll function to create an inclinometer display alongside the compass display. The inclinometer display guides users to position the phone 40° or 130° from nadir, for the water and sky images, respectively. The exact angle of the phone is saved in the HydroColor metadata when each image is captured. Therefore, the metadata can be used to ensure the images were captured at the correct angle.

### 2.2. Extracting Radiance from Images

**Figure 2:**
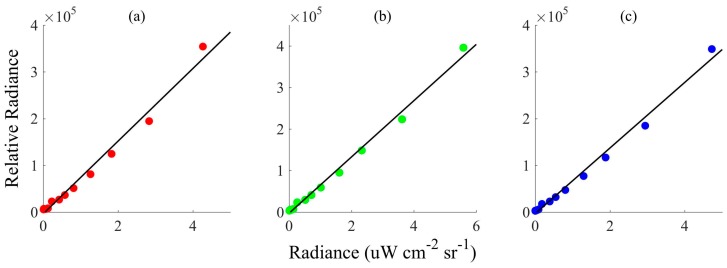
Camera response as a function of radiance. Measurements were made by gradually attenuating a white light source while collecting images with an Apple iPod touch. Relative radiance was calculated using Equation (2). The true radiance was measured using a Satlantic HyperPro radiometer. The response in the: red (**a**); green (**b**); and blue (**c**) color channels are shown. This figure is meant to show the linear relationship between the camera’s measure of relative radiance and the true radiance; it is not meant to provide an absolute calibration for measuring radiance.

A digital smartphone camera, while not meant to be a quantitative environmental sensor, can be used to measure light intensity. Most smartphone cameras use a complementary metal-oxide semiconductor (CMOS) as the light sensor. In the HydroColor application, light intensity is extracted from compressed images captured by the camera. Ideally, raw data from the CMOS sensor should be used. However, due to limitations of the standard application program interface (API) of common smartphones cameras, compressed image data are used. Compression introduces some nonlinearity into the camera response. However, it was found that light measurements were not greatly influenced by image compression (Figure 2). HydroColor measures relative light radiance using the following equation:

(2) $$L_{rel}=\;\frac{DN}{\alpha S}$$

where *DN* is the digital pixel value (0–255) from the camera image in either the red, green, or blue channel, α  is the exposure time, and *S* is the ISO speed. Aperture does not appear in the equation since the aperture is fixed for standard smartphone cameras. White balance of the camera does not appear because it is automatically locked by the HydroColor application at the beginning of each image collection sequence. Equation (2) is applied separately to each RGB channel to determine the relative radiance in each of the three channels.

To compare HydroColor measurements from different devices, the spectral sensitivity of the RGB channels must be similar between devices. Sensitivity curves for each device were acquired by recording the digital pixel values (0–255) returned by the camera while viewing the variable wavelength light source of a Cary 500 spectrophotometer. Light output from the spectrophotometer was varied from 400 to 700 nm at 1 nm intervals. A diffuser was placed between the camera and the light source to prevent pixel saturation. The exposure time, ISO speed, and white balance of the cameras were held constant during the spectral sensitivity measurement. The digital pixel values were recorded onboard the smartphone using a custom program. The resulting pixel values were smoothed using a 20 nm running average, then normalized by the largest recorded value in each channel.

### 2.3. Calculation of Remote Sensing Reflectance

Once the images are collected, they are processed onboard the phone to provide the remote sensing reflectance. To decrease the uncertainties of the measurements, the RGB pixel values from a 200 × 200 pixel region at the center of the image are averaged. The position of this region is highlighted when measurements are taken so that the user can minimize interferences by shadows and clouds. The average values are used to calculate the relative radiance using Equation (2). The relative radiances are used in Equation (1) to calculate the remote sensing reflectance.

As discussed previously, any scaling factor cancels out in Equation (1). Therefore, the camera needs no absolute calibration. All that is needed to calculate *R_rs_* is a quantity proportional to radiance. The proportionality between the actual radiance and the camera’s measure of relative radiance is likely to change with cleanliness of the lens, age, temperature, and device manufacturer. However, because the three images are all taken using the same device in a short period of time (less than a minute typically), these errors cancel out in the calculation of *R_rs_*.

### 2.4. Measuring SPM from Remote Sensing Reflectance

The concentration of suspended particulate matter (SPM) in the surface water has a direct and nonlinear effect on reflectance. SPM represents the concentration of all suspended particles, including organic and inorganic particles. Water is typically the dominating absorber in red wavelengths, leaving the red reflectance to be governed mostly by SPM concentration (via scattering). Chlorophyll does have an absorption peak at 676 nm, however, this peak falls nearly outside the red sensitivity curve of the camera. Indeed, both red and near infrared wavelengths are often used for satellite SPM algorithms [15,16]. Similarly, the change in reflectance associated with increasing SPM concentration can be detected in the HydroColor *R_rs_* red channel.

### 2.5. Field Tests

Field data were collected in three different campaigns. The first campaign was designed to compare HydroColor measurements to precision radiometers and turbidimeters for a wide range of water types. The second campaign was a volunteer driven HydroColor collection day, to demonstrate HydroColor as a citizen science tool. The third campaign was a brief comparison of HydroColor measurement from Android and Apple devices.

In the first campaign in 2013, field data were collected at three locations: the Columbia River mouth, the coast of Maine, USA, and the coast of Georgia, USA. Data were collected during the summer months under a variety of meteorological conditions, ranging from clear sky to fully overcast. An Apple iPhone 4, iPhone 5 or iPod touch where used to collect HydroColor measurements. Measurements were made under calm conditions where wind speed was less than 5 m s^−1^. The measurements were made in coastal case II waters where CDOM and mineral particles greatly influenced the water leaving radiance. Coastal Georgia measurements contained the highest amounts of mineral particles compared to the other study sites. Nearly all the field sites were influenced by freshwater input. HydroColor data were collected in parallel with an above water radiometer and a turbidimeter. The concurrent data were used to validate HydroColor’s calculation of *R_rs_* and provide a calibration for the measurement of turbidity.

A total of 63 HydroColor measurements collected during the first campaign were accompanied by measurements with a Water Insight Spectrometer (WISP). Both HydroColor and the WISP are designed to measure *R_rs_*. using the same method. The WISP uses three spectrometers to measure *L_t_*, *L_s_* and E_d_. Therefore, the WISP served as a convenient instrument to determine the accuracy of HydroColor. To ensure the most accurate results, the WISP was vicariously calibrated to agree with recently calibrated Satlantic Hyper-Pro radiometers. 

**Figure 3:**
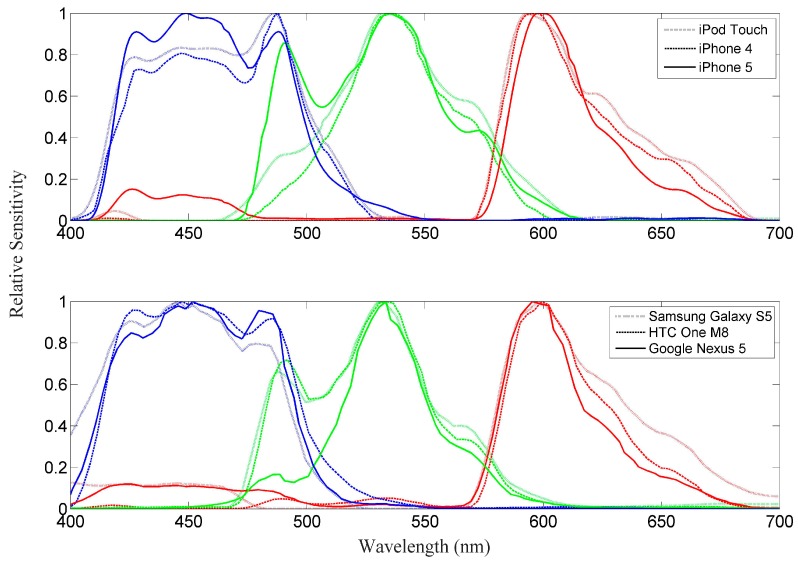
Spectral sensitivity curves for a variety of smartphone cameras. The red, green, and blue curves correspond to the red, green, or blue color channels of the camera. Each curve was smoothed using a 20 nm moving average, and then normalized by the highest recorded value.

*R_rs_* was calculated from WISP data using Equation (1), where E_d_ replaced the denominator. To compare the WISP hyperspectral *R_rs_* with HydroColor broadband *R_rs_*, the WISP spectra were averaged using the spectral sensitivity curves in Figure 3 as weights:

(3) $$R_{rs}\left(i\right)=\;\frac{\int_{400}^{700}R_{rs}\left(\lambda \right)w_{i}\left(\lambda \right)d\lambda }{\int_{400}^{700}w_{i}\left(\lambda \right)d\lambda }$$

where *R_rs_*(*λ*) is the *R_rs_* value measured by the WISP at wavelength *λ*, *w_i_*(*λ*) is the relative camera sensitivity in color channel *i* at wavelength *λ* (from Figure 3), and *R_rs_*(*i*) is the *R_rs_* value that would result if the WISP had the same spectral sensitivity as color channel *i*.

In this study, we used turbidity measured in nephelometric turbidity units (NTU) as a proxy for SPM concentration. Turbidity was measured using a Hach 2100Q portable turbidimeter. Using turbidity simplified the methodology and was a convenient choice since it is used by many environmental monitoring agencies. The turbidity dataset is made up of 58 different measurements collected on an iPhone 5, iPhone 4 or iPod touch. The turbidity measurements were made at the same study sites as the radiance measurements.

The second field campaign consisted of HydroColor measurements collected by volunteer citizen scientists. San Francisco Bay National Estuarine Research Reserve members organized volunteers to collect HydroColor measurements and water samples on 24 July 2014. In total, 14 measurements were collected along the shores of San Francisco Bay. Volunteers were spread over a large area of the bay spanning a wide range of turbidities. It is likely the measurements also spanned a wide range of chlorophyll and CDOM concentrations. SPM concentrations of the collected water samples were measured by filtration and gravimetric analysis. Sky conditions were partly cloudy.

The third field campaign was a device comparison between Apple iOS and Android based smartphones. This comparison was conducted at several sites around the Seattle, WA, USA area on 21 May 2017. HydroColor measurements from an iPhone 4, iPhone 5, iPhone 6, HTC One M8, Nexus 5 and a Galaxy S5 where collected sequentially at five different field sites. The field sites ranged from low turbidity water in Lake Washington and Puget Sound, to moderately turbid waters of the Duwamish and Puyallup rivers. Sky conditions were clear. The same gray card was used for all measurements, unlike the previous two campaigns. The reflectance and turbidity values measured by each device were compared to ensure HydroColor results were similar between a wide range of devices and operating systems.

## 3. Results

### 3.1. Camera Spectral Sensitivity

Spectral sensitivity curves from an iPod Touch, iPhone 4, iPhone 5, Samsung Galaxy S5, HTC one M8 and a Google Nexus 5 are shown in Figure 3. Note that this measure of sensitivity is for the camera as a whole, which includes the transmission of the Bayer and infrared filters and the sensitivity of the CMOS sensor itself.

The peak near 490 nm in the green response of several cameras appears to be an artifact. Typical Bayer filters used in color cameras do not have multi-peaked transmission spectra. The peak near 490 nm is most likely caused by cross talk between green and blue pixels [17]. The close alignment of the secondary green peak and the first blue peak provide further evidence that green pixels were, in some cases, influenced by optical or electrical cross talk from blue pixels.

Besides the artifact in the green curves, the spectral responses between all six cameras are very similar. These results are not surprising, as a significant deviation from these curves would not produce an acceptable color image to the human eye. Therefore, the spectral sensitivity can be assumed, for our purposes, to be device independent.

### 3.2. Validation of Reflectance Measurement

Equation (3) was applied to the WISP field data using the spectral sensitivity curve (Figure 3) from the camera used to collect the concurrent HydroColor measurement. For each color channel, a regression line was fit to the WISP and HydroColor *R_rs_* using type-I least-squares linear regression. After the initial fit, the standard deviation of the absolute error between the models predicted values and the measured values were calculated. Any data falling more than 3.5 standard deviations outside the median fit error were considered outliers and were removed from the regression. The regression line was then fit to the dataset with the outliers omitted. 

**Figure 4:**
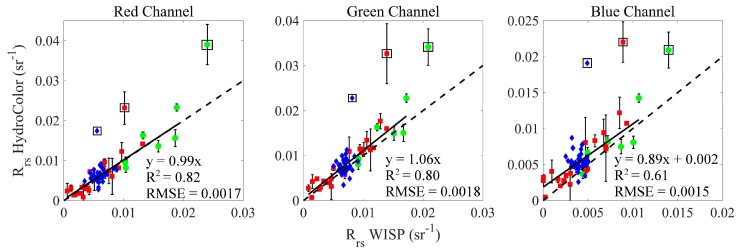
Comparison of HydroColor *R_rs_* with WISP *R_rs_*. The three plots show the *R_rs_* comparison for each color channel. To show a meaningful comparison, the WISP spectra were averaged using the camera spectral sensitivity curves as weights Equation (3). For replicate measurements, error bars display the standard error. The dashed line shows the one-to-one line and the solid line shows the results of a type-I linear regression. The data are broken out by location: Columbia River (blue triangles), Georgia (green circles), Maine (red squares). The data points in boxes were identified as outliers (using an objective procedure) and were not included in the regression.

The HydroColor measured *R_rs_* compares well with concurrent WISP measurements (Figure 4). The majority of data points fall close to the one-to-one line. The scatter of the data around the one-to-one line is similar for all channels (note the difference in scale for the blue channel in Figure 4). The range of *R_rs_* values spanned in the data set represent a wide range of conditions. However, HydroColor has yet to be tested in highly turbid waters (turbidity >80 NTU). The median percent error of the HydroColor measurement of *R_rs_* relative to the WISP was 18%, 16% and 26% for the red, green, and blue channels, respectively. The median bias for the same comparison was 1.3 × 10^−4^ sr^−1^, −5.2 × 10^−4^ sr^−1^, and−9.5 × 10^−4^ sr^−1^, respectively. The error in *ρ* is not realized in this comparison because the same value of *ρ* is used for both the HydroColor and WISP calculations of *R_rs_*.

There are several possible circumstances that may have generated the three outliers seen in Figure 4. The total duration of capturing the three images may play an important role, especially when the sky conditions exhibit patchy clouds. If the illumination or sky conditions change during the collection of the three images, it would lead to incorrect *R_rs_* values.

To further investigate HydroColor’s accuracy when measuring *R_rs_*, each component of Equation (1) can be looked at individually. When interpreting these data, it is important to note they are relative radiance values prior to taking any ratio. Therefore, they may contain biases due to white balance and variations in sensitivity. However, their examination can still provide useful conclusions.

**Figure 5:**
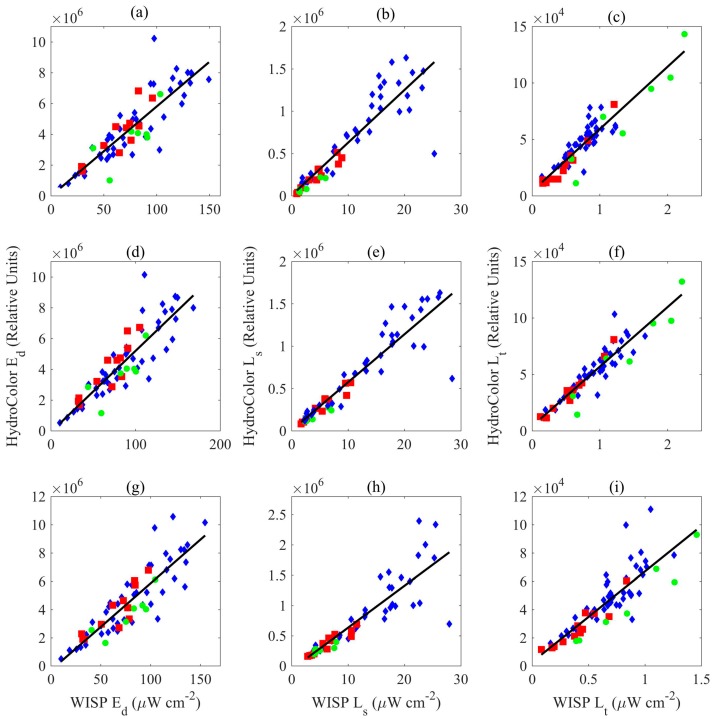
Comparison of HydroColor and WISP measurements of E_d_, *L_s_*, and *L_t_*. This figure shows data from the: red (**a**–**c**); green (**d**–**f**); and blue (**g**–**i**) color channels of the camera. Statistics are listed in Table 2. The data are broken down by device: iPod (green circles), iPhone 4 (red squares), and iPhone 5 (blue diamonds).

Figure 5 helps to confirm the assumption that any calibration factor needed to convert the camera measured relative radiance (*L_rel_*) to true radiance cancels out in the ratio of Equation (1). The slope values for E_d_, *L_s_* and *L_t_* are similar within each channel (Table 2). This also indicates that the conversion from the relative radiance of the gray card to the relative downwelling irradiance is correct. Figure 5 also shows that the smartphone cameras used in this study have similar sensitivities to light, however, this is not required for the method to work.

HydroColor’s measurement of E_d_ is more variable than the other two radiance measurements (Table 2). The gray card was always placed in a low area relative to the camera so the picture could be taken at a downward angle, possibly causing parts of the sky to be shaded by the person taking the image. The WISP irradiance sensor was typically at eye level. Variability in the reflectance of different gray cards could have also contributed. It is not known if age or long term sun exposure has an effect on gray card reflectance. These results suggest that HydroColor’s measurement of E_d_ is the largest source of error.

**Figure 6:**
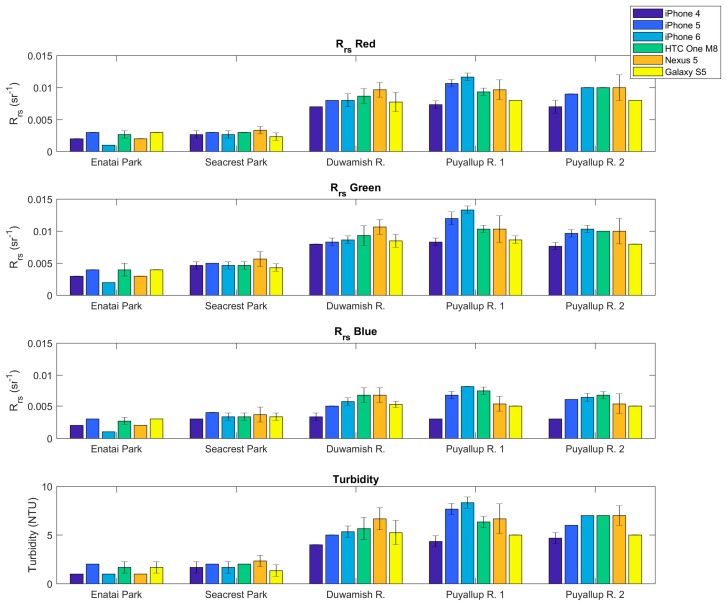
Comparison of the measured reflectance and turbidity from several smartphones at the same study sites. Bars show the mean of three measurements. Error bars show the standard deviation. No error bars present indicates the standard deviation between replicates was zero.

Data collected on 21 May 2017 using multiple Apple and Android devices allowed for an inter-device comparison of reflectance and turbidity. The study sites ranged from relatively clear water in Lake Washington (~2 NTU) to moderately turbid water of the Puyallup River (~7 NTU). Figure 6 show the reflectance and turbidity measured by each device at each study site. Only small variations in reflectance and turbidity exists between the six devices tested. None of the devices showed a consistent bias that was greater than the uncertainty of the HydroColor measurement.

### 3.3. Validation of Turbidity Measurement

**Figure 7:**
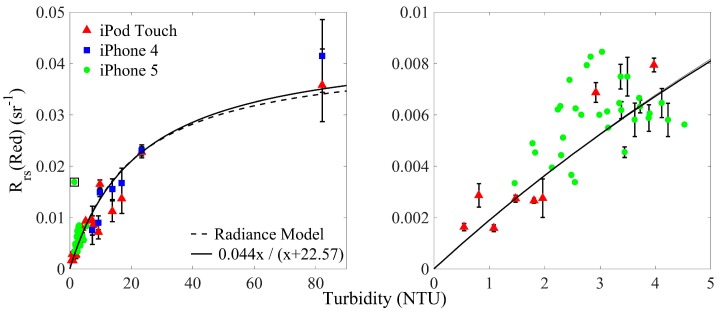
Relationship between turbidity and HydroColor R_rs_(Red). The plot on the right is a closer view of the lower turbidity values in the left plot. Error bars display the standard error (when replicate measurements were available). The single boxed data point identifies an outlier that was not included in the fitting of the radiance model or regression line. The solid lines and dash lines show the fitted relationships described in the validation section. The radiance model and regression line are on top of each another in the right plot. For the solid line R^2^ = 0.93 and RMSE = 0.003 sr^−1^.

The relationship between turbidity and HydroColor measured *R_rs_* in the red channel is shown in Figure 7. A function was fit to the data that provided an equation for converting HydroColor R_rs_ (Red) to turbidity. When applying this equation ($turbidity=\;22.57R_{rs}\left(Red\right)\;/\;\left(0.044-R_{rs}\left(Red\right)\right)$) back to the data in Figure 7, the median percent error in retrieval of turbidity was 24%. Compared to other methods for measuring turbidity [18], this accuracy is more than sufficient to collect meaningful turbidity measurements in inland and coastal waters.

To determine if the relationship between turbidity and R_rs_ (Red) is reasonable, an oceanic radiance model from Gordon et al. [19] was fit to the data. To fit the model, the relationship between turbidity and SPM concentration was assumed to be 1 NTU:1 g m^−3^ [18,20]. Fitting the radiance model to the turbidity and R_rs_ (Red) data provided a mass specific backscattering (b_bp_*) value of 0.010 m^2^ g^−1^ and a mass specific absorption (a_p_*) value of 0.0086 m^2^ g^−1^. These values are the effective b_bp_* and a_p_* values for the red channel of the camera, which spans a large portion of the visible spectrum. If a flat mass specific backscattering spectrum is assumed [21], the fitted b_bp_* provides the magnitude of the mass specific backscattering spectrum. A value of 0.010 m^2^ g^−1^ is within the range of values seen in the environment for the red portion of the visible spectrum [20].

The fitted value for a_p_* is slightly harder to interpret since the mass specific absorption spectrum of particles tends to be an exponentially decreasing function of wavelengths. However, using the iPod red sensitivity curve, an example of an absorption spectrum that would provide the value of 0.0086 m^2^ g^−1^ after a weighted average is: $a_{p}^{*}=0.015e^{-0.01\left(\lambda -550\right)}$. Both the magnitude and slope of this function are within the values seen in the environment [22,23]. Therefore, the relationship between turbidity and HydroColor R_rs_ (Red) is consistent with other published works. The error between the modeled and measured R_rs_ (Red) values is likely caused by the errors in *ρ* and variations in b_bp_* and a_p_* that are undoubtedly present in such a spatially large dataset.

### 3.4. Validation of Citizen Collected Data

Turbidity from volunteer collected measurements was derived from HydroColor reflectance using the relationship in Figure 7. Only the HydroColor derived turbidity was collected from volunteers, the metadata was not recovered. Therefore, it was not possible to determine if the volunteer collected measurements were performed correctly.

**Figure 8:**
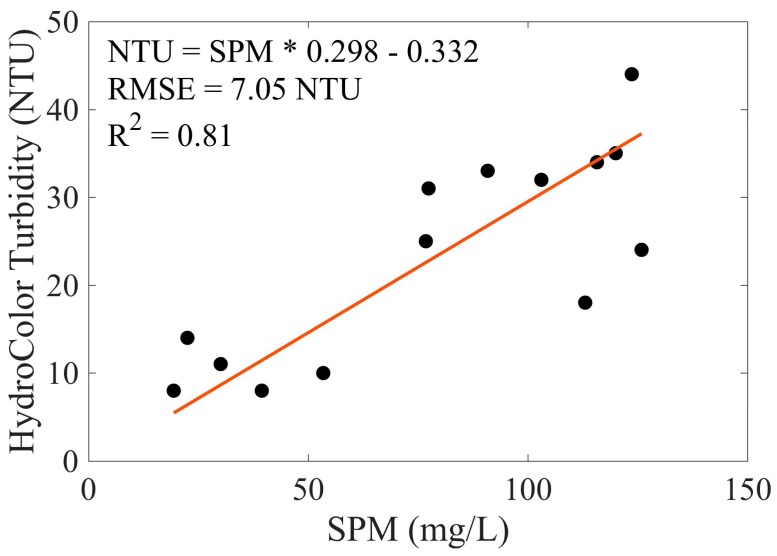
Comparison of volunteer collected HydroColor measurements and SPM in San Francisco Bay. SPM was derived from volunteer collected water samples. Red line and embedded statistics show a type-II linear regression.

SPM of the collected water samples and the turbidity reported by HydroColor were correlated (Figure 8). However, the relationship is much weaker than what is seen in Figure 7. This is likely due to errors in the measurement that went undetected, or differences in the absorption or scattering properties of the sediment at different location in the bay. The relationship of SPM and turbidity is also highly dependent on particle size and composition [18]. Even though this example demonstrates the worst-case scenario, where no HydroColor metadata were available for quality control. It shows that citizen scientists are able to observe trends in the environment using the HydroColor application.

## 4. Discussion

This study has demonstrated how a digital camera can be used as an aquatic remote sensing device. Smartphones are particularly well suited for this application because they contain a multitude of additional sensors. The HydroColor application can measure *R_rs_* in the red, green, and blue portions of the visible spectrum within 26% of a precision radiometer. Using the RGB *R_rs_*, various water quality parameters can be inferred. This study demonstrates that turbidity can be estimated, however, users can use the RGB reflectance to develop their own regional or global algorithms. While we do not think that a global algorithm for chlorophyll can be derived from HydroColor (e.g., using the green/blue reflectance ratio), due to variability of CDOM, it may be possible to derive a local algorithm that will work at a specific time and location.

HydroColor’s ability to measure reflectance accurately will depend on environmental conditions. Sea surface roughness can have a large effect on the value of *ρ*. When the surface of the water is sloped, an observer or detector is viewing a different portion of the sky than what is seen on a level surface. Currently HydroColor does not obtain any information on wind speed or sea state. Therefore, this is a known source of error in the calculation of *R_rs_*. The sensitivity of *ρ* to wind speed is a function of sun zenith angle and sky condition. As the sun is higher in sky, the influence of wind speed on *ρ* becomes greater. At a sun zenith angle of 30° and a wind speed of 15 m s^−1^, *ρ* can be as large as 0.05 [12]. This can lead to substantial error in *R_rs_*. Therefore, it follows that HydroColor should not be used in windy conditions where the sea surface is choppy. For purposes of this study, measurements were made in calm conditions and the value of *ρ* was assumed to be constant at 0.028. This is an accurate value of *ρ* for wind speeds less than approximately 5 m s^−1^. We observed that the most accurate data were collected under perfectly clear or homogeneously overcast skies, the cases modeled by Mobley [12]. See Appendix A for additional tips on using HydroColor.

It should be noted that inaccuracies in *R_rs_* due to sky conditions and a never truly known *ρ* value are not unique to HydroColor measurements. We have shown that a smartphone camera is able to measure a quantity proportional to radiance. Thus, the difficulties in minimizing surface reflectance and determining a correct *ρ* value for the calculation of *R_rs_* are inherent to even the most well calibrated above water radiometers systems.

Several improvements to the HydroColor application remain to be included. A large improvement to the application would be a better estimate of uncertainty. For example, examining the variability in an image, or in a series of images, could provide more information about meteorological conditions. The image variability could be an indicator of cloud cover or water surface roughness. This could be used to alert the user when conditions are not suitable for HydroColor measurements. Another improvement would be to make the sea surface reflectance factor (*ρ*) dynamic. This value could be adjusted based on results of image processing or weather data collected via the phone’s data connection. The use of raw camera data, instead of compressed data, would improve HydroColor’s ability to measure relative radiance. This feature will likely be implemented in the near future as new smartphone software and hardware features become available. Lastly, the HydroColor application uses a fixed number of pixels at the center of each image. Smartphones cameras have a wide variety of resolutions. Therefore, using a fixed area instead of a fixed number of pixels would help ensure the field of view of the camera is the same between devices.

HydroColor has the potential to make a large impact on both scientific and educational communities. Before the release of this application, such a simple and low cost method for measuring above water reflectance (and the derived turbidity) did not exist. The HydroColor application now allows citizen scientists to collect objective water quality data without the need for water samples, expensive instruments, or upfront time commitments. The data collected by HydroColor would complement already existing crowdsourcing efforts as well as ocean color remote sensing measurements. When coupled with Secchi Disk measurements and measurement using the Forel–Ule scale, for which mobile applications and cheap sensors exists [24,25,26], HydroColor can provide a different, and human-eye independent measurement of the water quality. Since all three are dependent on slightly different water properties, together they can better constrain water optical properties such as scattering and absorption. They can be used in concert as a quality check, as we would expect blue water based on Forel–Ule scale to have a high blue reflectance in the HydroColor application.

HydroColor has the additional advantage that the measurement tool and method are standardized across the globe. Smartphones are found worldwide, and they predominately contain hardware similar to what is described in this study. Assuming HydroColor is used correctly (which can be confirmed by examining the measurement metadata), the measurement method will have a high degree of consistency.

HydroColor also has value as an educational tool. Many students already own smartphones. Therefore, the infrastructure for wide scale educational application of HydroColor is already in place. HydroColor would be well suited as a teaching tool in environmental monitoring, oceanography, limnology, optics, and remote sensing classes. HydroColor can also be used as a public outreach tool, as the application can show people how a tool as simple as their camera can help them learn about their environment.

## Figures and Tables

**Table 1:** Abbreviations and Notations.

Symbol	Definition, Units
α	Exposure time, sec.
a_p_*	Mass specific absorption, m^2^ g^−1^
b_bp_*	Mass specific backscattering, m^2^ g^−1^
CDOM	Colored dissolved organic matter
DN	Digital pixel number (0–255)
E_d_	Downwelling irradiance, W m^−2^
*L_c_*	Gray card radiance, W sr^−1^ m^−2^
*L_rel_*	Relative radiance measured by a camera
*L_s_*	Sky radiance, W sr^−1^ m^−2^
*L_t_*	Water surface radiance, W sr^−1^ m^−2^
RGB	Red, Green, Blue
RMSE	Root mean squared error
*ρ*	Sea surface reflectance factor
*R_ref_*	Irradiance reflectance of a reflectance standard, unitless
*R_rs_*	Above water remote sensing reflectance, sr^−1^
S	ISO speed
SPM	Suspended particulate matter
WISP	Water Insight Spectrometer

**Table 2:** Statistics for type-I linear regressions in Figure 5.

Figure	Property	Channel	Slope (×10^4^)	Intercept (×10^4^)	R^2^	RMSE (×10^4^)
5a	E_d_	Red	5.82	−1.95	0.75	109.13
5b	*L_s_*	Red	6.23	0.22	0.84	19.90
5c	*L_t_*	Red	5.52	0.39	0.84	0.96
5d	E_d_	Green	5.27	−8.23	0.76	108.41
5e	*L_s_*	Green	5.67	0.47	0.86	17.93
5f	*L_t_*	Green	5.27	0.41	0.86	0.89
5g	E_d_	Blue	6.17	−31.76	0.76	114.82
5h	*L_s_*	Blue	6.92	−6.01	0.79	25.69
5i	*L_t_*	Blue	6.43	0.27	0.72	1.14

## Appendix A

Clear sunny skies or homogeneous cloud cover are the best conditions for using the HydroColor application. The sun zenith angle should be between 15 and 60 degrees. The measurement should be conducted in an open area with minimal shading by nearby objects. The measurement must be conducted in optically deep water. Since the water depth and clarity are typically unknown, the user must estimate if a location is deep enough. As a general rule of thumb, if any portion of the bottom is visible, this area is too shallow to collect HydroColor measurements. Docks, piers, and ships provide the best platforms for making HydroColor measurements. It is also important to avoid taking the water image in the shadow of a dock or any other structure.

HydroColor saves the reflectance, turbidity, and auxiliary data in a text file on the phones file system. The text file contains headers that describe the data. The data file can be offloaded from the device by connecting the smartphone to a computer. For Apple devices, the file can be downloaded from the application file-sharing tab in iTunes. For Android devices, the file system can be accessed using a free program such as Android File Transfer. The data file is stored in the HydroColor application folder.

The procedure utilized by HydroColor can be applied to almost any digital camera. When applying this method outside the HydroColor application, several things must be considered. It is important the white balance of the camera remains static when collecting images. This is accomplished in the HydroColor application by locking the white balance when the gray card image is collected. The application forces the user to collect the gray card image first so that all images are collected using the same white balance setting. Additionally, exposure and ISO speed of the camera must not be locked between the capture of images. This is important, since the sky is typically several times brighter than the water. Allowing the exposure and ISO speed to change, then compensating for the changes in Equation (2) increases the dynamic range of the relative radiance measurement.

Cameras can also be deployed facing in a fixed direction. The sun position will need to be tracked and the appropriate *ρ* value then applied to each series of image captures. This method could also be used if images are captured from an aerial platform where the viewing direction cannot be controlled. A table of *ρ* values based on wind speed and sun position can be found on the Ocean Optics Web Book website [27]. It should be noted that the directions provided here are intended to minimize surface glint and hence the uncertainty in its correction.
